# Referring patients to specialists: A structured vignette survey of Australian and British GPs

**DOI:** 10.1186/1471-2296-9-2

**Published:** 2008-01-15

**Authors:** Moyez Jiwa, Michael Gordon, Hayley Arnet, Hooi Ee, Max Bulsara, Brigitte Colwell

**Affiliations:** 1 Western Australian Centre for Cancer and Palliative Care, Curtin University of Technology, Perth Western Australia, Australia; 2 Gleadless Medical Centre, Gleadless, Sheffield, UK; 3 Sir Charles Gairdner Hospital, Perth, Western Australia, Australia; 4 University of Western Australia, Perth, Western Australia, Australia; 5 University of Sheffield, Sheffield, UK

## Abstract

**Background:**

In Australia and in the United Kingdom (UK) access to specialists is sanctioned by General Practitioners (GPs). It is important to understand how practitioners determine which patients warrant referral.

**Methods:**

A self-administered structured vignette postal survey of General Practitioners in Western Australia and the United Kingdom. Sixty-four vignettes describing patients with colorectal symptoms were constructed encompassing six clinical details. Nine vignettes, chosen at random, were presented to each individual. Respondents were asked if they would refer the patient to a specialist and how urgently. Logistic regression and parametric tests were used to analyse the data

**Results:**

We received 260 completed questionnaires. 58% of 'cancer vignettes' were selected for 'urgent' referral. 1632/2367 or 69% of all vignettes were selected for referral. After adjusting for clustering the model suggests that 38.4% of the variability is explained by all the clinical variables as well as the age and experience of the respondents. 1012 or 42.8 % of vignettes were referred 'urgently'. After adjusting for clustering the data suggests that 31.3 % of the variability is explained by the model. The age of the respondents, the location of the practice and all the clinical variables were significant in the decision to refer urgently.

**Conclusion:**

GPs' referral decisions for patients with lower bowel symptoms are similar in the two countries. We question the wisdom of streaming referrals from primary care without a strong evidence base and an effective intervention for implementing guidelines. We conclude that implementation must take into account the profile of patients but also the characteristics of GPs and referral policies.

## Background

In Australia, as in the United Kingdom (UK), patients can only access specialists after referral by a General Practitioner (GP) but notwithstanding these similarities there are marked differences in health care organisation. In Australia, there are two health care systems:

▪ A clearly defined public hospital system manages the health care needs of the entire population. Access to hospital specialists follows referral from GPs;

▪ A private hospital system manages patients with private health insurance, currently estimated to cover 44% of the population [1]. Access is also via referral by GPs however appointments in most cases are usually offered much more speedily than in the publicly funded hospital sector.

In Britain there are also two health care systems:

• A universally available and 'free at the point of use' National Health Service. Paid for by general taxation and used by the overwhelming majority of the population;

• A small private health care sector with only 11% of the population covered by health insurance [2].

Where cancer enters a GP's differential diagnosis, rapid access to specialists in the England is guaranteed for patients with signs and symptoms outlined in government guidelines [3]. In Australia no such arrangements exist and the scheduling of appointments to a publicly funded hospital is governed by local specialists based on the clinical details relayed in a referral letter. Whatever the context, it is generally acknowledged that there are substantial gaps between the best evidence and the management patients receive from doctors [4].

Colorectal cancer is an important public health problem globally with nearly one million new cases diagnosed world-wide each year and half a million deaths [5]. The best known symptoms of this cancer, namely persistent diarrhoea and episodes of rectal bleeding, are also common although the vast majority of people who experience such symptoms do not have cancer. In most cases a diagnostic colonoscopy is indicated to rule out malignancy rather than to confirm the clinical impression of cancer. However in recent years researchers suggest that it may be possible to identify those who are at higher risk of cancer from their symptom profile. Most colorectal cancers are diagnosed in patients over 65 years of age, in other words older than those currently eligible for government funded colorectal screening in Australia or the UK. Therefore the majority of cases will be diagnosed after medical consultation with symptoms rather than picked up at screening while asymptomatic. Few symptomatic people with lower bowel symptoms in Australia or Britain consult a medical practitioner and very few of these are referred for further investigation and definitive diagnosis to a specialist [6]. Of those symptomatic people who consult a GP, most can be managed without specialist advice but others are overlooked who would benefit [7,8].

It has been suggested that there is a wide variation in the way Australian GPs manage colorectal symptoms and by corollary, this may disadvantage some patients [9]. Data from the UK also suggest that cancer referral guidelines do not appear to have changed referral practice in Britain or outcomes for cancer patients [10]. In this structured vignette survey of GPs we aim to explore the impact of a variety of clinical and respondent characteristics on GPs' decision to refer patients with lower bowel symptoms.

## Methods

### Ethics

Ethics approval was granted in Australia by the ethics committee of the University of Western Australia (Ref: RA/4/1/1462) and in the UK by the North Sheffield LREC (NS 2004 2 1861). Return of the completed questionnaire was considered consent to participate in the survey.

A self-administered postal survey was carried out. Self-administered questionnaires have the advantage of dealing better with sensitive issues where anonymity is assured. Vignettes also capture something approximating a real-life situation and allow some degree of contextualization when considering the issues [11]. The value of this approach is also that we removed the confounding effect of doctor-patient miscommunication. Vignettes or 'stories' about patients with colorectal symptoms were constructed to include six clinical details with two possible variations. Therefore there were 64 potential scenarios to cover each of the possible combinations [12]. The vignettes were presented to the sample in an 'incomplete-within-blocks' design to reduce the number of vignettes presented to each respondent to nine.

Scenarios for 'cancer' patients were based on features of colorectal cancer as incorporated in UK national clinical guidelines and reflect guidance to Australian GPs [13,14]. Respondents were told that the patient in question did not have private health insurance in order to explore differences in decision making focusing on clinical rather than organisational issues. This implied the patient would have to be referred to a 'public' hospital where in both countries the patient would incur no charges and the appointment would be subject to the clinical need. Each respondent was potentially presented with two cases in which the evidence recommends an urgent referral:

1. Unexplained iron deficiency anaemia

2. Patient aged greater then 60 years of age with rectal bleeding and or altered bowel habit of at least 6 weeks duration. Although the UK guidelines stress the importance of looser or more frequent bowel motions as the significant change in bowel habit there has been doubt cast that persistent constipation is not a presenting symptom of cancer [15].

Thirty-eight out of the 64 vignettes contained at least one such scenario, henceforth referred to as 'cancer' vignettes. The other vignettes described cases that the guidelines imply do not constitute high risk cancer patients.

**Figure 1:**
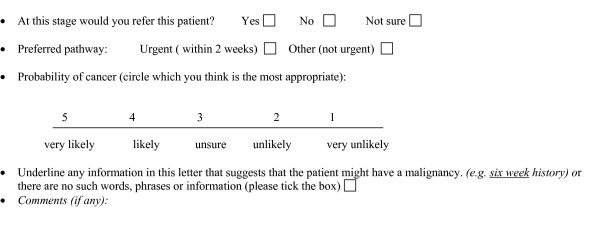
Examples of vignettes.

The questionnaire was piloted and refined following feedback from clinician volunteers. Each scenario was presented as a letter from a GP to a hospital specialist and styled in the manner displayed in the examples shown in Figure 1. Six clinical variables with two possible variations were included:

1. Age of patient (35 years or older than 60 years)

2. Duration of symptoms (6 weeks or two weeks)

3. Rectal bleeding (present or absent)

4. Change in bowel habit (present or absent)

5. Weight loss (present or absent)

6. Iron deficiency anaemia. (present or absent)

Each scenario contained similar information but was worded differently in order to introduce freshness to each case.

**Figure 2:**
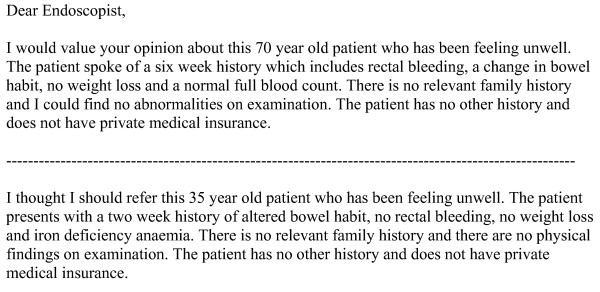
Questions posed after each vignette.

The respondents were asked whether they would refer the patient described in each scenario, how soon they would seek investigation and if they thought the patient may be harbouring a malignancy as shown in Figure 2.

Demographic data about each respondent collected included:

1. Age

2. Sex

3. Number of patients seen per week.

4. Single-handed/group

5. Number of years in clinical practice

6. Teaching status

7. Geographical location (Metro, outer metro and rural). Outer metropolitan areas are urban centres with populations of around 100,000 people. Rural areas are those serving populations of less than 25,000 [16].

In order to model up to 13 explanatory variables for one outcome variable we required 154 respondents [17]. We anticipated a 50–60% response rate. Logistic regression was used to examine the relative importance of specific signs, symptoms and risk factors that may influence the decision to refer via the urgent or 'other' pathway. In order to control for clustering, a cluster option was used to estimate robust standard errors. All analysis was performed on Stata V9 [18].

### Recruitment

GPs were recruited at random from the list of practitioners registered at the Royal Australian College of General Practitioners (RACGP) in Western Australia and in the UK from GPs listed in metropolitan areas of North Trent. In Australia recruitment was also designed to include a significant number of rural practitioners. Questionnaires were disseminated by post with a covering letter. Where necessary a reminder questionnaire was sent six weeks after the first mailing. Anonymity was protected, as practitioner identities were coded with only administrative staff able to identify individuals.

## Results

Five hundred questionnaires were distributed, two-hundred and sixty completed responses were received, a response rate of fifty-two percent. The genders of the respondents were equally distributed with the majority of practitioners older than 40 years of age. In Australia approximately one third of respondents were rural practitioners, which corresponds to the known distribution of practitioners in that country [19]. Most practitioners had been in practice more than five years and the majority were in group practice and were teaching practices. The demographic details of the respondents are shown in Table 1. The 'correct' decision to refer patients on the 'appropriate' pathway (i.e. 'urgent' referral for those with cancer symptoms and routine referral for all other cases) was made in 56.2% of cases overall. 'Cancer vignettes' were presented 1433 times in the survey. On three hundred and fifty occasions (25%) cases were explicitly recognised as 'likely' or 'very likely' to have cancer but 775 (58%) were referred 'urgently'.

**Table 1:** Demography of respondents to survey

	**SITE**		
**Variable**	**UK**	**WA**	**Total**
GP Gender			
Males	46 (51.7)	87 (50.9)	133 (51.1)
GP Age Group			
20–29	0	10 (5.8)	10
30–39	21 (23.6)	59 (34.5)	80
40–49	35 (39.3)	55 (32.2)	90
50–59	25 (28.1)	30 (17.5)	55
60+	7 (7.9)	17 (9.9)	24
Practice Location			
Rural	0	52	52
Metro	89	79	168
Outer Metro	0	41	41
Number of years in practice			
<5	8	40	48
5–10	17	29	46
10–15	16	32	48
15–20	16	25	41
20+	31	45	76
Full time equivalent GPs in the practice			
<=1	15	21	36
2–4	45	82	127
5–7	27	54	81
>=8	2	11	13
Teaching			
None	28	49	77
Medical students	17	51	68
GP Registrars	12	12	24
Other	7	6	13
Combinations	25	51	76

### Vignettes selected for referral

1632 or 69% of the vignettes were selected for referral. The outcome variable: 'refer now' was set as the independent variable and modelled with six respondent variables as explanatory variables including: location of practice (rural, metro and outer metro), site of practice (UK vs. WA), respondent age and gender, number of years in practice and teaching status along with the clinical variables in the vignettes namely age of patient described in vignette, gender, rectal bleeding, change in bowel habit, iron deficiency anaemia and weight loss. After adjusting for clustering the model suggests that 38.4% of the variability is explained by the independent variables. The model has a sensitivity of 94.12% and a specificity of 41.78%. The positive predictive value is 78.89% and negative predictive value of 75.45%. Table 2 displays only those variables which significantly contributed to the model. Area under the ROC curve was 0.89 (95% CI: 0.87, 0.91). Practicing in Australia as opposed to the UK was not found to be relevant. All six clinical variables and the age of the respondent and length of clinical experience were significant.

**Table 2:** Impact of variables on decision to refer symptomatic patient

**Variable**	**OR**	**Robust S.E.**	**P>|z|**	**95% C.I.**
**Respondent variables**				

Respondent age				
30–39	5.3	2.72	0.001	1.9 to 14.5
40–49	3.2	1.74	0.03	1.1 to 9.3
Years in practice (20+)	3	1.24	0.007	1.3 to 6.8

**Vignette variables**				

Age in vignette (> 60 years)	2.95	0.46	<0.001	2.2 to 4.0
Duration of symptoms (6 weeks)	2.3	0.38	<0.001	1.7 to 3.2
Rectal bleeding	11.76	2.99	<0.001	7.1 to 19.4
Altered bowel habit	4.94	0.84	<0.001	3.5 to 6.9
Iron Deficiency anaemia	7.12	1.28	<0.001	5.0 to 10.2
Weight loss	2.62	0.46	<0.001	1.9 to 3.7

### Vignettes referred 'urgently'

1012 or 42.8 % of vignettes were referred 'urgently', of these 911 vignettes (90%) merited an 'urgent' referral based on the criteria mentioned above. The outcome variable: 'refer urgently' was set as the independent variable and modelled with six respondent variables as explanatory variables including; location of practice (rural, metro and outer metro), site of practice (UK vs. WA), respondent age and gender, number of years in practice and teaching status along with the clinical variables in the vignettes namely **age of patient described in vignette, gender, rectal bleeding, change in bowel habit, iron deficiency anaemia, weight loss **and cancer vignette as defined by clinical guidelines. After adjusting for clustering, the data suggests that 31.3 % of the variability is explained by these variables. The model has a sensitivity of 81.7% and a specificity of 65.6%. The positive predictive value is 68.6% and negative predictive value is 79.6%. Table 3 displays the extent to which variables significantly contributed to the model. Area under the ROC curve was 0.86 (95% CI: 0.83, 0.87). 'Cancer vignettes' were twice as likely to be selected for urgent referral as others. All the clinical variables listed in bold above were significant and only the age of the respondent and practicing in outer metro locations were significant.

**Table 3:** Impact of variables on decision to refer patients urgently

**Variable**	**OR**	**Robust S.E.**	**P>|z|**	**95% C.I.**
**Respondent variables**				

Outer metro location	2.2	0.76	0.01	1.2 to 4.4
30–39	2.8	0.87	0.001	1.5 to 5.2
40–49	2.3	0.91	0.03	1.1 to 5.0
50–59	4.6	2.12	0.001	1.9 to 11.4
60+	15.4	9.84	<0.001	4.4 to 53.8

**Vignette variables**				

Age in vignette (> 60 years)	2.9	0.4	<0.001	2.4 to 4.4
Duration of symptoms (6 weeks)	2.1	0.28	<0.001	1.6 to 2.7
Rectal bleeding	4.4	0.73	<0.001	3.2 to 6.1
Altered bowel habit	3.5	0.51	<0.001	2.8 to 4.8
Iron Deficiency anaemia	2.1	0.50	<0.001	2.5 to 4.5
Weight loss	4.1	0.61	<0.001	2.9 to 5.3
'Cancer vignette'	2.0	0.5	0.01	1.2 to 3.40

## Discussion

The differences in health care organisation were not reflected in a difference in GP decisions about which patients to refer when focused on publicly funded services in these countries. Assuming that a colonoscopy is warranted for unexplained iron deficiency anaemia or for persistent symptoms in an older patient, just over half the cases presented in this study would be referred on the 'correct' pathway and a slightly greater proportion of 'cancer vignettes' selected for 'urgent' referral. However only a quarter of these, were explicitly recognised as warranting an urgent referral because of a higher risk of 'cancer', noting that 38 out of the 64 were intended to be 'cancer vignettes', indicating uncertainty when diagnosing cancer in a population where the condition presents infrequently. Interviews with GPs about managing lower bowel symptoms suggest that the mode of presentation and the disclosure of embarrassing symptoms had a significant influence on the decisions made at consultation [20]. By presenting clinicians explicitly with clinical details we were able to record decisions not influenced by miscommunication. Surprisingly and despite any possible respondent bias, the data are consistent with practice recorded in a review of actual referrals in the locality where this study was conducted in the UK [21]. Ideally we would have liked to validate clinician responses to this survey with observations from their clinical practice but this was not possible within the resources at our disposal.

A reasonable degree of variability was explained by the clinical and respondent variables modelled. The symptom of weight loss exerted considerable influence on the decision to refer patients urgently. Weight loss is a common feature of many cancers and is often a harbinger of poor prognosis [22]. Therefore practitioners may be selecting patients for urgent investigation when the disease is already at a relatively advanced stage. There is some evidence that colorectal cancer patients referred from general practice to public hospitals in Western Australia enter specialist services with advanced disease [23]. Evidence from the UK also suggests guidelines have made little difference to the prognosis of colorectal cancer patients in that country where they are promoted to stream patients for urgent specialist appointments [24]. The impact of 'morbidity' has previously been demonstrated as misleading practitioners at the point of referral. Mitchell and colleagues concluded that referrals to specialists were sometimes based on measures that did not correspond with serious pathology [25]. More recent evidence in studies of colorectal cancer suggests that weight loss is not a marker of advanced disease [26]. However, in the context of upper gastrointestinal cancer weight loss is accepted as indicating poor survival. Therefore patients with so-called 'alarm symptoms' and incurable pathology may be selected for urgent endoscopy while those lesser morbidity but treatable pathology channelled to routine waiting lists [27]. Therefore weight loss is not a sensible reason for referral if early diagnosis of cancer is the goal. That is not to say that patients with marked weight loss do not warrant an early specialist appointment, but weight loss, on current evidence is not a helpful basis on which to refer patients for timely treatment of gastrointestinal cancers.

The influence of patients' characteristics on referral decisions has been widely reported and echo much of the data reported here. In their study of differences in referral rates in the UK Sullivan et al conclude that 'morbidity' is likely to explain some of the variability but much of the variability remains 'unexplained' [28]. However a study of Canadian family practitioners also explored practitioner characteristics and similarly posit that the age and practice location of the referring doctor exert an appreciable impact on referral patterns even within Canada's universal health insurance system [29]. In our data relating to Australian practice these factors were also of interest although with small numbers and no other explanatory data we can only speculate on the reasons.

To politicians, health care managers and the general public guidelines promote evidence based practice, protect patient safety, ensure equity of access and or contain costs. However compliance with existing referral 'guidelines' is a contentious issue. In respect to colorectal cancer, recent research calls into question the value of 'early' referral of patients with 'symptomatic' colorectal cancer. 'Delay' for patients with the recognised 'red flag' symptoms measured in months or up to a year may not inevitably result in a poorer prognosis [30]. The issue of 'red flag' symptoms was also challenged in a systematic review that concluded that clinical features currently listed in guidelines have unacceptably low predictive value [24]. Focusing on practitioner decision making Gabbay and May suggested that doctors do not act on published evidence base but on the anecdotes and unsubstantiated opinions of respected colleagues or personal experience [31]. Our data support the notion that the application of guidelines is moderated, or even negated, by the characteristics of those for whom they are intended. This is important because, in Britain, GPs are involved in prioritising health care expenditure in a so-called 'primary care led' National Health Service and serve as both poacher and game keeper [32].

## Conclusion

In this structured vignette study selection of patients with colorectal symptoms reflects the data from surveys of actual referrals and does not indicate consistently evidence based practice. However despite these similarities in referral behaviour the observed outcomes for colorectal cancer are moderately better in Australia than they are in the UK [33]. We speculate as others have done that this may be because GPs have greater access to urgent colonoscopy in Australia, perhaps via the private health care sector [23]. We conclude that practitioners should have access to rapid appointments for those patients whose symptoms are worrisome either because of morbidity or because the patient may benefit from curative therapy. This calls into question the wisdom of streaming referrals from primary care without effective interventions for the implementation of evidence based guidelines.

## Competing interests

The author(s) declare that they have no competing interests.

## Authors' contributions

MJ conceived of the study and the design. MJ drafted the manuscript. MG coordinated the study in the UK. HE contributed to the design of the study and the preparation of the manuscript. HA and BC entered the data and contributed to the preparation of the manuscript. MB analysed the data. All authors read and approved the final manuscript.

## Pre-publication history

The pre-publication history for this paper can be accessed here:


